# Feasibility and Impact of a Coached Home-Based Exercise Program on Cardiorespiratory Fitness in Adults With Cystic Fibrosis on Highly Effective Modulator Therapy

**DOI:** 10.1016/j.chpulm.2026.100252

**Published:** 2026-03-09

**Authors:** Ashley Barry, Peter Niedbalski, Allison Bustos, Dave Burnett, Jianghua He, Joel Mermis

**Affiliations:** a Respiratory Care and Diagnostic Science, University of Kansas Medical Center, Kansas City, KS; b Pulmonary and Critical Care, University of Kansas Medical Center, Kansas City, KS; c Internal Medicine, University of Kansas Medical Center, Kansas City, KS; d Department of Biostatistics and Data Science, University of Kansas Medical Center, Kansas City, KS

**Keywords:** adherence, airway clearance, barriers to exercise, cardiorespiratory fitness, cystic fibrosis, Cystic Fibrosis Questionnaire-Revised, elexacaftor/tezacaftor/ivacaftor, highly effective modulator therapy, home-based exercise, hyperpolarized xenon-129 MRI scan, Incremental Shuttle Walk Test, personalized exercise, physical activity, quality of life, ventilation defect percent

## Abstract

**Background:**

In the era of highly effective modulator therapy (HEMT), people with cystic fibrosis (PwCF) are living longer and experiencing a shift in disease burden, with exercise emerging as an essential strategy to support cardiorespiratory and metabolic health. Despite known benefits, adherence to exercise remains low, and evidence guiding implementation of home-based interventions is limited. This study assessed the feasibility and clinical impact of a coached home-based exercise program in PwCF on HEMT.

**Research Question:**

Does a personalized, coached home-based exercise program improve cardiorespiratory fitness in PwCF who are on stable HEMT?

**Study Design and Methods:**

This randomized controlled pilot study enrolled PwCF on stable elexacaftor/tezacaftor/ivacaftor. Participants were randomized to a 12-week coached home-based exercise intervention or standard care. Intervention participants received weekly coaching and progressed to 180 min/wk of moderate-intensity exercise. Primary outcome was change in cardiorespiratory fitness measured by the Incremental Shuttle Walk Test. Secondary outcomes included adherence, changes in lung function (FEV_1_ % predicted), health-related quality of life (Cystic Fibrosis Questionnaire-Revised), perceived barriers to physical activity, and ventilation defect percent measured by hyperpolarized xenon-129 MRI scan.

**Results:**

Twenty-one PwCF were enrolled, with 14 participants completing the study (intervention: n = 8; control: n = 6). The intervention group demonstrated a significant improvement in Incremental Shuttle Walk Test distance compared with the control group (76.3 ± 98.4 m vs −66.7 ± 93.3 m, respectively). The between-group difference favored the intervention by 14.3 m (95% CI, 2.96-25.63; *P* = .03). No significant changes were observed in FEV_1_ % predicted, Cystic Fibrosis Questionnaire-Revised total score, barrier scores, or ventilation defect percent.

**Interpretation:**

Our results show that a personalized, coached home-based exercise program significantly improved cardiorespiratory fitness in PwCF on HEMT but had no measurable impact on lung function or quality of life. These findings support the feasibility of a home-based exercise coaching program.

**Clinical Trial Registration:**

ClinicalTrials.gov; No.: NCT05239611; URL: www.clinicaltrials.gov

**Take-Home Point**

**Study Question:** Does a personalized, coached home-based exercise program improve cardiorespiratory fitness in adults with cystic fibrosis who are on stable highly effective modulator therapy?

**Results:** Personalized home-based exercise program led to improvements in cardiorespiratory fitness but did not measurably impact lung function or quality of life.

**Interpretation:** As the care model for cystic fibrosis continues to evolve, implementation of a home-based exercise program may offer an effective strategy to improve cardiorespiratory fitness outcomes.

The proportion of adults living with cystic fibrosis (CF) relative to the overall CF population increased from 29% to 60% over the last 3 decades and is expected to continue to increase.^1^ This increased life expectancy poses new challenges for people with cystic fibrosis (PwCF) whose direct effects of their disease often coupled with lifestyle modifications, such as inactivity, lead to exercise intolerance. Lack of exercise in turn leads to low cardiorespiratory fitness, which is a strong, independent predictor of morbidity and mortality in PwCF.^2^ A meta-analysis reported a nearly 5-fold increased risk of mortality for PwCF with low cardiorespiratory fitness, measured as maximal oxygen volume (Vo_2_max).^3^

Current literature clearly indicates that exercise has a positive impact in PwCF, and that clinicians consider exercise an important therapeutic strategy for improving the health of PwCF.^4^ Studies have found that increasing physical activity levels and participation in exercise training programs have a positive impact on cardiorespiratory fitness, pulmonary function, muscle mass, and health-related quality of life (HR-QoL) in CF.^5^ Exercise is also associated with improved mental health and has benefits in glucose regulation and weight management, which have become increasingly relevant since the widespread use of highly effective cystic fibrosis transmembrane conductance regulator modulators (highly effective modulator therapy [HEMT]).^6^ Thus, in the HEMT era, PwCF and CF teams are wanting to better understand if exercise can replace traditional mucociliary clearance (MCC) techniques while also providing additional health benefits including cardiovascular fitness needed to mitigate risk of undesired weight gain and CF-related diabetes.

Exercise training is a common therapeutic strategy for outpatient treatment in CF. Although both patients and clinicians consider exercise important, clinicians have limited evidence-based strategies to promote the uptake of exercise interventions, and < 25% of adult patients with CF report adhering to prescribed exercise.^7^ To improve adherence, partially supervised home-based interventions have been used in PwCF. Home-based interventions have several advantages over fully supervised interventions, including reduced travel cost and time, ability to select a broad range of exercises based on personal preference, and the ability to incorporate exercise into a daily routine. However, the feasibility and efficacy of a home-based exercise program in PwCF is not known.

In this study, we evaluate the feasibility of a coached home-based exercise program and its impact on cardiorespiratory fitness, lung function, and HR-QoL. We hypothesize that exercise capacity, HR-QoL, and lung function will improve through exercise intervention.

## Study Design and Methods

This was a randomized controlled pilot and feasibility study of a home-based exercise program among PwCF. Participants were recruited from the University of Kansas Health System adult CF program. All study procedures were approved by the University of Kansas Medical Center Institutional Review Board (KUMC STUDY00146919) , and informed consent was obtained from all participants.

### Participants

Adults with CF (2 CF mutations or sweat chloride > 60 mmol/L) who were ≥ 18 years of age and stable on HEMT elexacaftor/tezacaftor/ivacaftor for ≥ 3 months were eligible to participate. Exclusion criteria are included in e-Appendix 1. Eligible participants who consented were randomized 1:1 via a computer-generated randomization technique to either the intervention or control group. Participant demographics, Incremental Shuttle Walk Test (IMSWT), spirometry, hyperpolarized xenon-129 MRI (Xe-MRI) scan, Cystic Fibrosis Questionnaire-Revised (CFQ-R), and surveys regarding physical activity were completed at baseline and study completion. Semistructured interviews evaluating exercise were completed in the intervention group at the conclusion of the follow-up visit. Although participants were not informed of their group allocation, the trial could not be masked because the difference between group content could not be concealed from participants or research assistants.

### Exercise Protocol

Those randomized to the intervention group were assigned to an exercise coach and asked to wear a wrist-worn Garmin Vivosmart 4 actigraph during physical activity. Participants were asked to complete 12 weeks of progressive home-based exercise in a target heart rate zone, with an exercise target of 60 minutes in weeks 1 and 2, 90 minutes in weeks 3 and 4, 120 minutes in weeks 5 through 8, and 180 minutes in weeks 9 through 12. Target heart rate zone was calculated using the heart rate reserve method for moderate activity levels as described by the American College of Sports Medicine, which uses an intensity factor of 40% to 85% of the maximal heart rate achieved during the IMSWT. Exercise coaches contacted participants weekly by phone to discuss strategies for reaching exercise goals and provide personalized feedback regarding exercise motivations and barriers. Physical activity was participant-determined and based on individual preferences, lifestyle, and resources. The method of physical activity was variable and included, among other strategies, obtaining a gym membership, attending structured classes, using home equipment (dumbbells, mini trampoline, or treadmill), sports (kayaking, hiking, or soccer), and outdoor activities (playing with pets, gardening, or neighborhood walking/jogging).

The control group received standard-of-care instructions regarding exercise and instruction on how to access beamfeelgood, an online repository of exercise videos available free of charge for PwCF.

### Outcome Measures

The IMSWT was used as a surrogate for cardiorespiratory fitness because it has shown construct validity and predictive value for Vo_2_max in adults with CF.^8^ Testing was conducted according to the protocol outlined by Singh et al. Distance and maximal heart rate were collected at the end of the IMSWT.

Adherence to exercise was calculated as the number of exercise minutes completed in the target heart rate zone each week/weekly target exercise minutes. Participants were considered adherent if they completed ≥ 70% of weekly exercise minutes for ≥ 70% of the 12-week intervention.

Spirometry was obtained at enrollment and completion of study. FEV_1_ was expressed as % predicted using The Third National Health and Nutrition Examination Survey (NHANES III) spirometric reference ranges.

The CFQ-R is a valid and reliable self-administered questionnaire composed of 44 items on 12 generic and disease-specific domains and is used to assess HR-QoL in adults with CF.^9^ Overall CFQ-R score and each domain score were collected.

The Barriers to Physical Activity questionnaire assesses barriers to participating in physical activity and asks participants to rank 15 items on a 5-point Likert scale from never to very often limiting physical activity. Overall barrier score and each item score were collected.

Xe-MRI scan was used to measure ventilation and gas exchange. Xe-MRI scan was completed ≥ 30 minutes before the IMSWT for all study participants. Isotopically enriched (90% ^129^Xe) xenon gas (Linde Specialty Gases) was polarized to > 25% using a Polarean 9820 xenon hyperpolarizer (Polarean Imaging Plc). The total dose volume delivered to participants was equal to 20% of FVC measured at the baseline study visit.^10^ Participants were imaged using a 3T Skyra MRI scanner (Siemens) using a flexible transmit/receive ^129^Xe vest coil (Clinical MR Solutions, LLC). Calibration data were analyzed in real time using a home-built MATLAB (version 2020A; MathWorks) program, and the outputs were used for subsequent imaging. After calibration, every participant was imaged using 3-dimensional ventilation imaging.^11^ Within the same breath, a geometry-matched ^1^H anatomic image set was acquired to be used for image quantification.

Xe-MRI images were quantified using an automated pipeline to quantify ventilation defect percent (VDP). VDP was calculated using the mean anchored linear binning approach with a threshold of 60%. VDP was an exploratory end point for this study.

### Data Analysis

To achieve 80% power at an α level of 0.05 (2-sided), 12 participants were needed in each group to detect an effect size of 1.2. A target enrollment of 15 per group (n = 30) was set to account for attrition.

Data are presented as mean ± SD, and categorical data are presented as a percentage. Baseline between-group differences were analyzed with a Wilcoxon-Mann-Whitney test for continuous variables and a Fisher exact test for categorical variables, as appropriate. No between-group differences were found for demographic or baseline measures. Group differences for all outcome measures were assessed using a Wilcoxon-Mann-Whitney test. An intention-to-treat approach was used for participant baseline characteristics. Participants with missing postintervention data were excluded from group difference analyses.

All statistical analyses were performed using SAS v9.4 (SAS Institute Inc). All comparisons were 2-sided with the level of significance set at *P* < .05.

## Results

**Figure 1:**
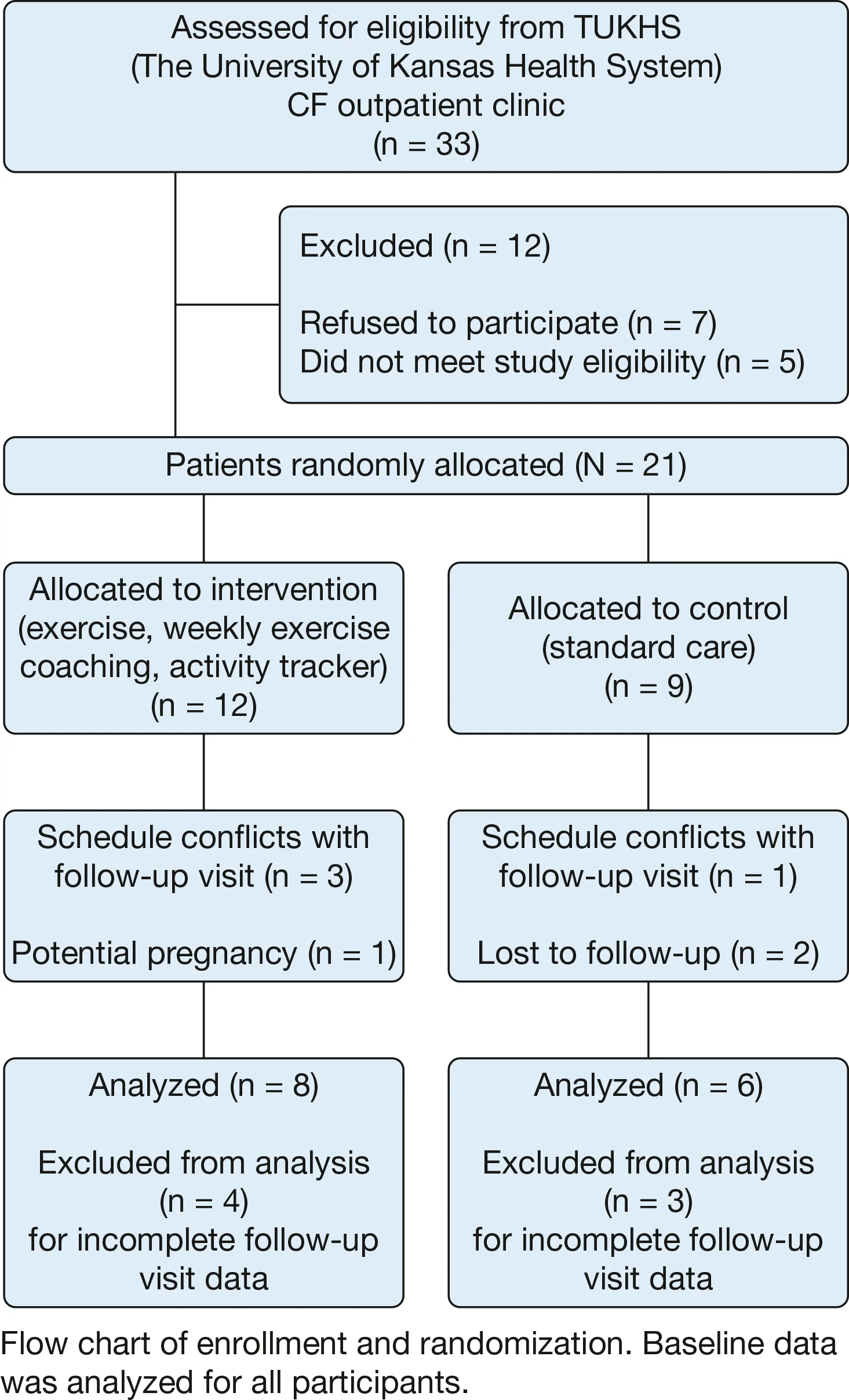
Consolidated Standards of Reporting Trials diagram. Flowchart of enrollment and participation. Baseline data were analyzed for all participants. CF = cystic fibrosis; TUKHS = The University of Kansas Health System.

Figure 1 shows the Consolidated Standards of Reporting Trials (CONSORT) diagram. Initial enrollment target was 30 adults (15 per group). Enrollment was impacted by a high prevalence of telehealth visits post-COVID-19 and prolonged time frames between clinic visits, resulting in early termination of recruitment efforts after enrollment of 21 individuals. Of the 21 participants, 6 in the control group and 8 in the intervention group completed prestudy and poststudy visit assessments, resulting in 33% attrition for both groups.

The baseline participant characteristics for the control group (n = 9) and the intervention group (n = 12) are shown in Table 1. The mean age ± SD of the study cohort was 30.66 ± 6.54 years (range, 21-49 years). In total, 8 participants (38%) were female. There were no significant differences between groups at baseline.

**Table 1:** Participant Baseline Characteristics

Characteristic	All Participants (N = 21)	Control Group (n = 9)	Intervention Group (n = 12)	*P* Value
Sex				
Male	13 (62)	8 (89)	5 (42)	.07
Female	8 (38)	1 (11)	7 (58)	
Age, y	30.66 [6.54]	28 [4.13]	33 [7.48]	.16
Race				
White or Caucasian Ethnicity	21 (100)	9 (100)	12 (100)	.99
Not Hispanic or Latino	21 (100)	9 (100)	12 (100)	.99
BMI, kg/m^2^	25.26 [3.45]	26.3 [3.48]	24.5 [3.35]	.24
Height, cm	168.7 [9.1]	170.2 [4.9]	167.6 [11.2]	.48
Weight, kg	72.3 [13.7]	76.8 [10.9]	68.9 [14.9]	.25
FEV_1_ % predicted	81.61 [17.04]	84.22 [15.53]	79.67 [18.52]	.45
IMSWT, m	788.1 [276.02]	898.89 [247.26]	705 [276.55]	.14
CFQ-R	81.2 [12.63]	82.57 [10.63]	80.17 [14.32]	.90
VDP, %	22.0 [10.4]	20.7 [9.0]	24.2 [12.6]	.49
Conventional airway clearance/MCC therapies				
None	8 (38)	2 (22)	6 (50)	.37
Vest high frequency oscillation	3 (14)	2 (22)	1 (8)	.55
Hypertonic saline	3 (14)	1 (5)	2 (10)	.99
Dornase alpha	8 (38)	4 (44)	4 (33)	.67
Inhaled antibiotic	5 (24)	3 (33)	2 (17)	.61

Data are presented as mean [SD], No. (%), or as otherwise indicated. CFQ-R = Cystic Fibrosis Questionnaire-Revised; IMSWT = Incremental Shuttle Walk Test; MCC = mucociliary clearance; VDP = ventilation defect percentage.

### Primary End Point

After the 12-week exercise program, the intervention group demonstrated improved cardiorespiratory fitness as measured by the IMSWT, with a mean increase ± SD of 76.3 ± 98.4 m, whereas the control group showed a mean loss of 66.7 ± 93.3 m. The between-group difference favored the intervention by 14.3 m (95% CI, 2.96-25.63; *P* = .03). Of those randomized to the exercise intervention, 7 of 8 participants (88%) had an increase in exercise capacity (Table 2).

**Table 2:** Change in Baseline Measures

Baseline Measure	Control Group (n = 6)	Intervention Group (n = 8)	*P* Value
FEV_1_ % predicted	1.2 [4.66]	−0.38 [3.42]	.34
IMSWT, m	−66.7 [93.3]	76.3 [98.4]	.03
CFQ-R overall	−2 [10.82]	−4.91 [7.06]	.42
Physical	−1.39 [18.76]	−8.33 [17.82]	.49
Vitality	−4.17 [20.24]	−15.63 [12.15]	.23
Emotion	7.78 [8.86]	−9.17 [10.04]	.01
Eat	0 [14.05]	−8.33 [15.43]	.39
Treat	7.41 [16.73]	−8.33 [11.5]	.87
Health	−7.4 [9.07]	−6.95 [8.27]	.99
Social	−1.85 [9.73]	0 [11.11]	.69
Body	5.56 [11.65]	−8.33 [15.43]	.16
Role	0 [13.94]	−8.33 [11.78]	.27
Weight	−22.22 [34.43]	−4.17 [11.78]	.22
Respiratory	−19.19 [27.54]	−4.17 [12.86]	.99
Digest	3.7 [18.14]	−9.72 [13.85]	.16
VDP, %	0.18 [2.0]	0.09 [4.15]	.95

Data are presented as mean [SD] or as otherwise indicated. CFQ-R = Cystic Fibrosis Questionnaire-Revised; IMSWT = Incremental Shuttle Walk Test; VDP = ventilation defect percentage.

### Secondary End Points

Two of the 8 intervention group participants (25%) who completed the poststudy visit achieved successful adherence to the exercise program as assessed via the activity monitor-recorded exercise minutes. Four of the 6 participants who did not achieve adherence reported technical issues with the activity monitor or forgot to wear the monitor, resulting in ≥ 3 weeks per participant with no exercise data recorded. Using self-reported exercise minutes, 50% of participants in the intervention arm (n = 4) who returned for the follow-up visit achieved successful adherence.

Change in overall CFQ-R between groups was not statistically significant (*P* = .42). Interestingly, of the group randomized to exercise, 50% of participants had lower emotion domain scores at follow-up, resulting in a significant change in this CFQ-R category compared with the control group (*P* = .01).

Barriers to completing regular physical activity were assessed at baseline and study completion. Change in barriers was not statistically significant. Lack of time was the primary barrier at baseline and follow-up for both groups. e-Table 1 shows the change in barriers to physical activity.

Change in FEV_1_ % predicted between groups was not statistically significant (*P* = .34).

**Figure 2:**
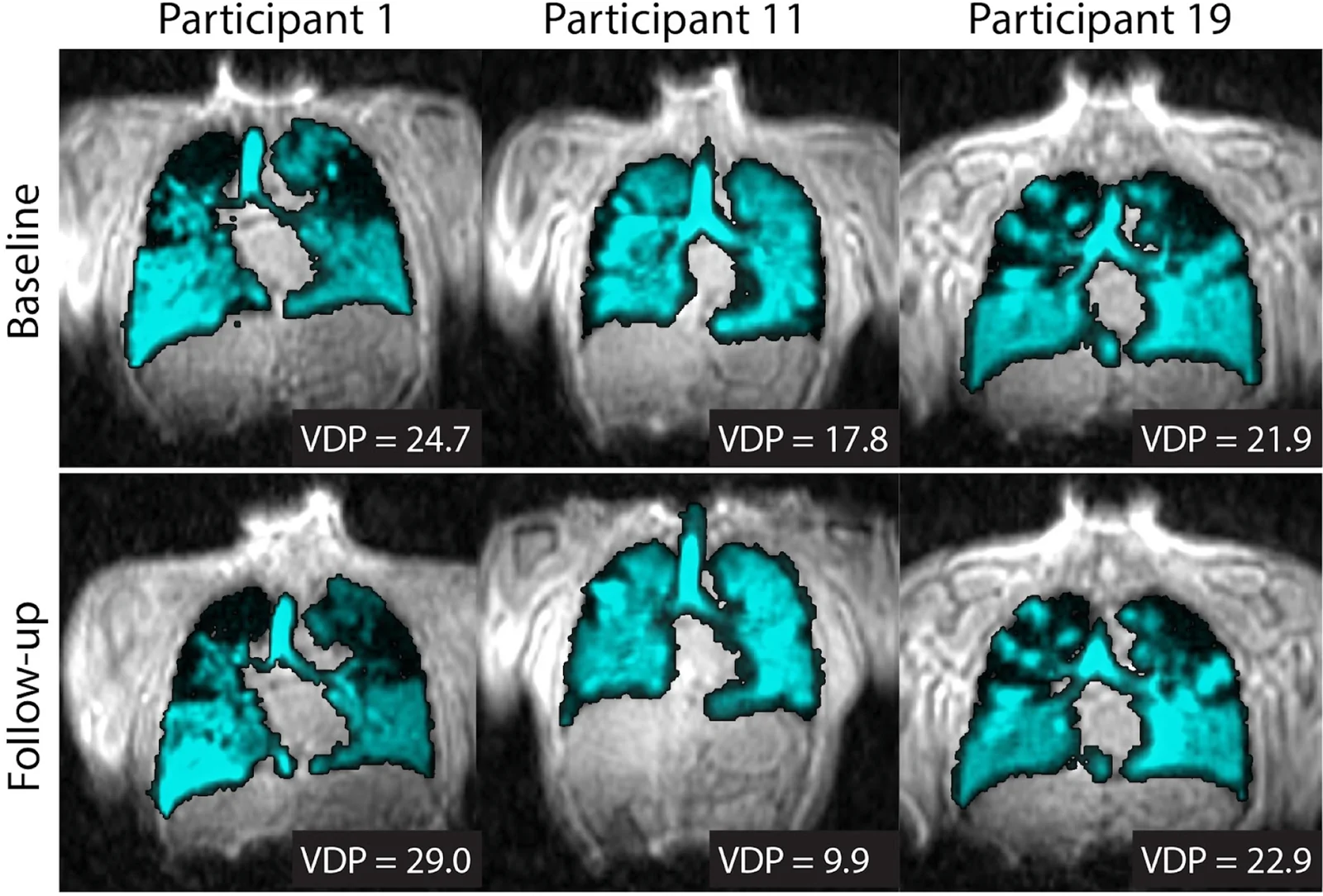
Representative hyperpolarized xenon-129 MRI ventilation images for 3 participants at baseline and follow-up. Although some participants had significant changes to ventilation (eg, participant 11), most participants had minimal change in ventilation with similar VDP and size/location of ventilation defects across time points. VDP = ventilation defect percent.

At baseline, Xe-MRI imaging results were consistent with known imaging features in the CF population.^12^ Specifically, participants carried a high burden of ventilation defects (VDP, 22.0% ± 10.4%), and ventilation defects showed a strong apical predominance. VDP at baseline was not significantly different between participants in the control and intervention arms (20.7% ± 9.0% vs 24.2% ± 12.6%, respectively; *P* = .49). Change in VDP from baseline to follow-up was not statistically significant in either group (control, *P* = .83; intervention, *P* = .95). Figure 2 shows the Xe-MRI images evaluating VDP.

## Discussion

### Primary End Point: Exercise Capacity

This study was designed to evaluate the impact of a 12-week coached home-based exercise program on clinical and patient-centered outcomes in PwCF on HEMT. Compared with participants randomized to standard of care exercise prescription in clinic, exercise coached participants who completed the follow-up assessment had an improvement in the primary outcome (exercise capacity as measured by the MSWT). Nearly all participants in the exercise group had improved exercise capacity compared with only 2 of 6 in the standard of care group (Table 3).

**Table 3:** Participant Demographics and Outcomes for Control and Intervention Groups

Participant	Gender	Age, y	Performs Daily Conventional Airway Clearance	ppFEV_1_		IMSWT, m		VDP, %		CFQ-R		BMI, kg/m^2^		Self-Report Adherence, %	Garmin Adherence, %	Comments
				Baseline	Follow-Up	Baseline	Follow-Up	Baseline	Follow-Up	Baseline	Follow-Up	Baseline	Follow-Up			
Intervention group																
1	Male	24	No	88	87	970	1,100	24.68	28.97	81.15	78.36	24.8	24.6	92	92	
2	Male	30	No	114	113	1,240	1,330	4.98	4.13	96.53	94.21	26.5	26.6	50	50	
3	Female	40	No	75	81	420	430	24.15	27.58	52.2	59.45	20.85	20.6	0	71	Did not wear actigraph for 5 wk of intervention
4	Female	49	Yes (vest daily)	49	NA	200	NA	43.36	NA	87.27	NA	27.7	NA	42	33	Withdrew after completing 12 wk of intervention; scheduling conflicts with follow-up visit
5	Male	35	No	82	81	750	900	12.68	11.73	93.29	82.43	23.8	23.9	29	57	Did not wear actigraph for 7 wk of intervention
6	Female	31	No	85	87	820	970	11.96	10.25	96.53	98.38	24.8	25	83	67	
7	Female	33	No	73	NA	580	NA	36.38	NA	94.58	NA	31.2	NA	100	100	Withdrew on week 4 for potential pregnancy
8	Male	37	No	44	NA	520	NA	45.93	NA	62.89	NA	20.4	NA	25	63	Withdrew on week 9 for time commitment reasons
9	Male	34	No	83	NA	780	NA	17.21	NA	67.13	NA	27.3	NA	92	75	Withdrew after completing 12 wk of intervention; scheduling conflicts with follow-up visit
10	Female	26	No	87	85	780	660	24.07	28.99	64.35	64	24	24	67	75	Admitted to the hospital within 48 h of follow-up visit for unrelated illness
11	Female	21	No	90	90	510	540	17.76	9.94	78.75	69.42	22.6	22.6	42	50	Actigraph malfunction
12	Female	31	No	86	80	890	1,060	26.97	26.38	76.32	64.54	19.8	19.6	100	89	Did not wear actigraph for 4 wk of intervention; shoelaces came untied during follow-up IMSWT
Control group																
13	Male	26	No	96	NA	1,060	NA	19.46	NA	89.25	NA	24.1	NA	NA	NA	Withdrew (lost to follow-up)
14	Female	34	No	88	NA	480	540	12.44	9.12	91.46	92.25	33.6	NA	NA	NA	Spirometry data unavailable; participant cancelled clinic visit after completing study follow-up visit
15	Male	24	No	76	78	940	810	30.98	32.42	72.1	62.78	21.6	21.9	NA	NA	
16	Male	25	No	89	NA	1,070	NA	18.89	NA	94.58	NA	28	NA	NA	NA	Withdrew due to scheduling conflicts with follow-up visit
17	Male	26	No	78	73	620	540	24.92	25.71	81.81	61.41	25.9	25.9	NA	NA	
18	Male	24	No	110	NA	1,210	NA	3.09	NA	71.2	NA	23.6	NA	NA	NA	Withdrew (lost to follow-up)
19	Male	34	Yes (vest daily)	92	92	1,130	950	21.89	22.87	93.89	92.22	24.8	24.8	NA	NA	
20	Male	28	Yes (vest daily)	56	64	840	740	32.46	31.59	66.18	74.12	28.3	27.7	NA	NA	
21	Male	32	No	73	74	740	770	22.26	24.33	82.62	88.26	26.8	30.8	NA	NA	

Data are presented as No. or %. CFQ-R = Cystic Fibrosis Questionnaire-Revised; IMSWT = Incremental Shuttle Walk Test; NA = not applicable; ppFEV_1_ = FEV_1_ % predicted; VDP = ventilation defect percentage.

### Secondary End Points: FEV_1_, Adherence, CFQ-R, Barriers to Exercise, and Lung Function Assessment Via Xe-MRI Scan

The detection of FEV_1_ improvement in PwCF on HEMT is likely to be challenging given the long-term stability of lung function assessed by spirometry.^13^^,^^14^ As anticipated, there was no significant difference in FEV_1_ % predicted between groups. Although our study was not powered to detect a difference in FEV_1_ % predicted, this finding supports the notion that a more sensitive measure of success of pulmonary intervention is needed. Because minimal change was expected in spirometry measures due to stability on HEMT, Xe-MRI scan was used as a novel strategy to assess lung function in PwCF.

Exercise data collection via the activity monitor in the exercise group was impacted by lack of consistent use of the device by some participants and technical issues for others. Given the more complete data set in comparison with activity monitor data, self-reported exercise adherence rates were much higher. Although self-reported exercise adherence often overestimates actual adherence, 5 of the 8 participants (63%) who attended the follow-up visit had higher total exercise time reported by their activity monitor than through self-report. Participants were more likely to report only structured, intentional exercise time, and the activity monitor algorithm would record exercise after 5 minutes of an elevated heart rate combined with motion, which may contribute to the discrepancy between self-reported and activity monitor exercise minutes.

Studies suggest that exercise can improve psychological health and sense of well-being. Interestingly, both the intervention and control groups demonstrated a decrease in their overall CFQ-R scores; however, this was not statistically significant. Emotional domain scores were lower during the follow-up visit for individuals in the intervention group and slightly higher for those in the control group. The lower domain scores among the intervention group conflicts with the themes found in the semistructured interviews conducted after the completion of the follow-up visit, where several participants reported that exercise had significantly improved feelings of anxiety and overall mood. This evidence should be treated with caution because these interviews were primarily conducted as quality improvement for exercise coaching and therefore not completed in the control group. Limited inferences can be drawn from this outcome measure due to the small sample size and the impact of COVID-19 acting as a potential confounder to emotion and sense of well-being.

Findings from this study are consistent with previous findings assessing perceptions of exercise before the approval of HEMT, with PwCF indicating that lack of energy, lack of self-discipline, and lack of time were the most common perceived barriers.^15^ Lack of good health was previously reported as a frequent barrier to physical activity but was reported as one of the lowest perceived barriers in our study, likely due to the impact of HEMT on overall health.

Although FEV_1_ has been the historic standard outcome for evaluating clinical efficacy of pulmonary interventions in CF, in the HEMT era, a more sensitive measure of lung function is needed.^12^ To address this issue, we used an emerging technique to assess impact of the exercise intervention on lung function: Xe-MRI scan.^16^ Baseline Xe-MRI findings across both the control and intervention groups demonstrated apical ventilation defects as anticipated in PwCF. We hypothesized that exercise-induced clearance on inspissated secretions would improve ventilation as measured by VDP. Although Xe-MRI scan has exhibited the ability to be a more sensitive and effective method for the longitudinal assessment of CF lung disease compared with FEV_1_, participants on HEMT in this study, regardless of exercise, demonstrated minimal change over 12 weeks in lung disease as measured by VDP.^17^ Xe-MRI scan has consistently demonstrated high sensitivity to lung function impairment in CF lung disease, including high sensitivity to improvements to function after initiation of HEMT. Notably, Xe-MRI scan appears to have greater sensitivity to lung function improvement than other common measures of lung function, including spirometry and lung clearance index. Furthermore, Xe-MRI scan is highly repeatable in PwCF.^18^ With this high sensitivity and repeatability, the lack of change in VDP observed in this study would suggest that regular exercise, despite myriad other benefits, may not improve lung function in PwCF on HEMT. At the same time, the small sample size requires caution in interpretation. Future study in larger patient populations will be needed. Although such studies using Xe-MRI scan as an outcome measure of airway clearance are challenging from logistical, budgetary, and technical perspectives, their feasibility is increasing with wider adoption of Xe-MRI scan, particularly at CF centers of excellence.

### Impact on CF Care

The care model for CF is rapidly changing because advances in treatment with HEMT have dramatically improved symptoms and life expectancy for most PwCF.^19^ As a result, the CF community has begun to evaluate the safety of reducing treatments, with the hopes of decreasing the burden of care. Results from the Discontinuation vs Continuation of Hypertonic Saline or Dornase Alfa in Modulator Treated People With Cystic Fibrosis (SIMPLIFY) study support the decision to reduce inhaled therapies used to facilitate MCC.^20^ Additionally, the percentage of individuals using no airway clearance or substituting exercise for airway clearance has grown since the approval of HEMT. In line with this trend, all but 3 participants in this study indicated at the time of enrollment they were performing no routine conventional airway clearance. Data demonstrating effectiveness of either conventional airway clearance or exercise for MCC in the HEMT era are lacking. However, because studies before HEMT demonstrate improvement in MCC associated with exercise, physical activity as an alternative to traditional airway clearance modalities in the HEMT era merits further study.^21^ Current conventional MCC modalities often impose a significant time burden and may contribute to the frequent report of time as a barrier to participating in physical activity in PwCF. Additionally, exercise may provide added benefits in mental health, cardiovascular health, and metabolism, and is further supported by the strong association between improved exercise capacity and survival in PwCF. The additional advantages of exercise may be especially important in the HEMT era, because 40% of adults with CF have diabetes, and the percentage of adults classified by the Centers for Disease Control and Prevention as overweight or obese has risen from 15.8% in 2002 to 41.4% in 2023.^22^

Lung function assessed via Xe-MRI scan did not demonstrate overall improvement in those randomized to the exercise intervention but was stable with consistent findings of stable FEV_1_ from longer-term study in PwCF on elexacaftor/tezacaftor/ivacaftor.^13^ Although most PwCF on HEMT have stable lung function, the US CF registry and other studies continue to demonstrate increasing prevalence of complications of metabolic syndrome including hypertension, dyslipidemia, and metabolic dysfunction-associated fatty liver.^23^, ^24^, ^25^ These findings raise concerns about a potential shift in morbidity and mortality among PwCF on HEMT, with complications of metabolic dysfunction emerging as a more prominent contributor, potentially more than respiratory disease for some. This is likely particularly true for those with normal or near-normal lung function such as in our study. Given this evolving phenotype, CF care should be personalized, with a greater emphasis on exercise for many PwCF.

Because PwCF are overall generally healthier, they are leading busy lives, often limiting time to participate in a formally structured exercise program. PwCF are more likely to adhere to an exercise regimen when they enjoy the activity and find it manageable in terms of time and cost. A strength of this study is its use of a progressive and individualized exercise approach, enabling participants to gradually increase exercise duration while using accessible resources to meet recommended levels.

Although we think these findings are relevant to all adult CF care teams, there are limitations in both data interpretation and program implementation. Inconsistent use of Garmin monitors and technical issues affected exercise data collection. Additionally, a higher-than-expected dropout rate partly due to the COVID-19 pandemic also limited data. Finally, our team had the resources and time to commit to a weekly coaching program for this limited cohort. We acknowledge that this level of commitment to a CF program’s entire patient population may not be feasible. Scaling this model may present challenges due to CF team demands and sustained personnel time. Scalable approaches worth future study would include hybrid models such as optimization of digital platforms or automated clinical support tools to help sustain the program and reduce the burden on clinicians and CF teams.

## Interpretation

In this study, a personalized, coached home-based exercise program led to improvements in cardiorespiratory fitness but did not significantly impact CF-related symptoms, as measured by the CFQ-R, or lung function, as assessed by Xe-MRI scan. As the CF phenotype continues to evolve in the HEMT era, larger studies are needed to assess the long-term effects of sustained exercise on cardiorespiratory fitness, pulmonary function, metabolic health, and symptom burden.

## Funding/Support

This project was supported in part by a biostatistics, epidemiology and research design (BERD) voucher award from the Frontiers Clinical and Translational Science Institute at University of Kansas Medical Center. Research reported in this publication was supported by the Frontiers Clinical and Translational Science Institute under award number UL1TR002366 from the National Center for Advancing Translational Sciences of the National Institutes of Health. The content is solely the responsibility of the authors and does not necessarily represent the official views of the NIH.

## Financial/Nonfinancial Disclosures

None declared.

## Supplementary Data

e-Online Data

## References

[1] Cromwell E.A., Ostrenga J.S., Todd J.V. (2023). Cystic fibrosis prevalence in the United States and participation in the Cystic Fibrosis Foundation Patient Registry in 2020. J Cyst Fibros.

[2] Hebestreit H., Hulzebos E.H.J., Schneiderman J.E. (2019). Cardiopulmonary exercise testing provides additional prognostic information in cystic fibrosis. Am J Respir Crit Care Med.

[3] Vendrusculo F.M., Heinzmann-Filho J.P., da Silva J.S., Perez Ruiz M., Donadio M.V.F. (2019). Peak oxygen uptake and mortality in cystic fibrosis: systematic review and meta-analysis. Respir Care.

[4] Gruet M., Saynor Z.L., Urquhart D.S., Radtke T. (2022). Rethinking physical exercise training in the modern era of cystic fibrosis: a step towards optimising short-term efficacy and long-term engagement. J Cyst Fibros.

[5] Radtke T., Smith S., Nevitt S.J., Hebestreit H., Kriemler S. (2022). Physical activity and exercise training in cystic fibrosis. Paediatr Respir Rev.

[6] Beaudoin N., Bouvet G.F., Coriati A., Rabasa-Lhoret R., Berthiaume Y. (2017). Combined exercise training improves glycemic control in adult with cystic fibrosis. Med Sci Sports Exerc.

[7] Rand S., Prasad S.A. (2012). Exercise as part of a cystic fibrosis therapeutic routine. Expert Rev Respir Med.

[8] Campos N.E, Vendrusculo F.M., DA Costa G.A., DE Almeida I.S., Becker N.A., Donadio M.V.F. (2022). The association of field test outcomes with peak oxygen uptake in patients with cystic fibrosis: a systematic review. Int J Exerc Sci.

[9] Quittner A.L., Sawicki G.S., McMullen A. (2012). Psychometric evaluation of the Cystic Fibrosis Questionnaire-Revised in a national sample. Qual Life Res.

[10] Niedbalski P.J., Hall C.S., Castro M. (2021). Protocols for multi-site trials using hyperpolarized (129) Xe MRI for imaging of ventilation, alveolar-airspace size, and gas exchange: a position paper from the (129) Xe MRI clinical trials consortium. Magn Reson Med.

[11] Niedbalski P.J., Willmering M.M., Thomen R.P. (2023). A single-breath-hold protocol for hyperpolarized (129) Xe ventilation and gas exchange imaging. NMR Biomed.

[12] Smith L.J., Horsley A., Bray J. (2020). The assessment of short- and long-term changes in lung function in cystic fibrosis using (129)Xe MRI. Eur Respir J.

[13] Bower J.K., Volkova N., Ahluwalia N. (2023). Real-world safety and effectiveness of elexacaftor/tezacaftor/ivacaftor in people with cystic fibrosis: Interim results of a long-term registry-based study. J Cyst Fibros.

[14] Nichols D.P., Paynter A.C., Heltshe S.L. (2022). Clinical effectiveness of elexacaftor/tezacaftor/ivacaftor in people with cystic fibrosis: a clinical trial. Am J Respir Crit Care Med.

[15] Burnett D.M., Barry A.N., Mermis J.D. (2020). Physical activity level and perception of exercise in cystic fibrosis. Respir Care.

[16] Thomen R.P., Walkup L.L., Roach D.J., Cleveland Z.I., Clancy J.P., Woods J.C. (2017). Hyperpolarized (129)Xe for investigation of mild cystic fibrosis lung disease in pediatric patients. J Cyst Fibros.

[17] Ratjen F.A., Stanojevic S., Munidasa S. (2025). Multicenter Study of Hyperpolarized Xenon Magnetic Resonance Imaging in Children with Cystic Fibrosis Following Initiation of Cystic Fibrosis Transmembrane Regulator Modulator Therapy (HyPOINT). Ann Am Thorac Soc.

[18] Walkup L.L., Roach D.J., Plummer J.W. (2025). Same-day repeatability and 28-day reproducibility of xenon MRI ventilation in children with cystic fibrosis in a multi-site trial. J Magn Reson Imaging.

[19] Brown R.F., Close C.T., Mailes M.G. (2024). Cystic fibrosis foundation position paper: redefining the cystic fibrosis care team. J Cyst Fibros.

[20] Mayer-Hamblett N., Ratjen F., Russell R. (2023). Discontinuation versus continuation of hypertonic saline or dornase alfa in modulator treated people with cystic fibrosis (SIMPLIFY): results from two parallel, multicentre, open-label, randomised, controlled, non-inferiority trials. Lancet Respir Med.

[21] Ward N., Morrow S, Stiller K., Holland A.E.. (2021). Exercise as a substitute for traditional airway clearance in cystic fibrosis: a systematic review. Thorax.

[22] Annual Data Report Cystic Fibrosis Foundation Patient Registry. https://www.cff.org/medical-professionals/patient-registry.

[23] Eldredge J.A., Oliver M.R., Ooi C.Y. (2024). Cystic fibrosis liver disease in the new era of cystic fibrosis transmembrane conductance regulator (CFTR) modulators. Paediatr Respir Rev.

[24] Gramegna A., De Petro C., Leonardi G. (2022). Onset of systemic arterial hypertension after initiation of elexacaftor/tezacaftor/ivacaftor in adults with cystic fibrosis: a case series. J Cyst Fibros.

[25] Poore T.S., Taylor-Cousar J.L., Zemanick E.T. (2022). Cardiovascular complications in cystic fibrosis: a review of the literature. J Cyst Fibros.

