# Supplementary material for: Feasibility and Impact of a Coached Home-Based Exercise Program on Cardiorespiratory Fitness in Adults With Cystic Fibrosis on Highly Effective Modulator Therapy
Source: CHEST Pulm. 2026 Mar 9;4(3):100252. doi: 10.1016/j.chpulm.2026.100252 (PMC13563564; doi:10.1016/j.chpulm.2026.100252)
Supplement: Supplementary file 1 [file mmc1.docx]

**Supplementary materials**

**Supplementary Methods:**

Exclusion criteria included completion of ≥ 150 minutes of structured exercise per week for the last three months, pregnancy, post solid organ transplant, active treatment for mycobacterial infections, oxygen dependent at rest or with exercise, forced expiratory volume in one second (FEV_1_)< 40% of predicted, clinical evidence of cor pulmonale, untreated arterial hypertension (resting systolic blood pressure >140 mm Hg, diastolic blood pressure > 90 mmHg), systolic blood pressure < 90 mm Hg while standing, congestive heart issues, active treatment for Allergic Bronchopulmonary Aspergillosis (ABPA), pulmonary exacerbation within the last four weeks, changes in therapy (including antibiotics) for pulmonary disease within the last four weeks, significant hemoptysis within 4 weeks (≥5 mL of blood in one coughing episodes or >30 mL of blood in a 24 hours period), and ongoing participation in an investigational drug study within the last 60 days.

**Supplementary Table 4.**

| Table 4. Barriers to Physical Activity | | | | | |
| --- | --- | --- | --- | --- | --- |
|  | Control (n=6) | | Intervention (n=8) | |  |
|  | Baseline | Change | Baseline | Change | p-value |
| Self-conscious about my looks when I exercise | 1 ± 0 | -0.17 ± 0.41 | 1 ± 1 | 0.13 ± 0.64 | 0.64 |
| Lack of interest in exercise or physical activity | 3 ± 1 | -0.33 ± 0.82 | 3 ± 1 | 0.38 ± 1.06 | 0.3 |
| Lack of self-discipline | 3 ± 1 | 0.33 ± 1.03 | 3 ± 1 | 0 ± 0.53 | 0.67 |
| Lack of time | 3 ± 2 | -0.67 ± 1.75 | 3 ± 1 | -0.13 ± 0.64 | 0.46 |
| Lack of energy | 3 ± 1 | -0.5 ± 1.05 | 3 ± 1 | 0.38 ± 0.74 | 0.16 |
| Lack of company | 1 ± 1 | 0.33 ± 0.52 | 2 ± 1 | 0.75 ± 0.89 | 0.52 |
| Lack of enjoyment from exercise or physical activity | 2 ± 1 | 0 ± 1.1 | 2 ± 1 | -0.13 ± 0.64 | 0.7 |
| Discouragement | 2 ± 1 | -0.33 ± 0.52 | 1 ± 0 | 0.25 ± 0.71 | 0.23 |
| Lack of equipment | 2 ± 1 | 0 ± 0.63 | 2 ± 2 | -0.5 ± 1.2 | 0.57 |
| Lack of good weather | 2 ± 0.9 | 0 ± 1.27 | 2.8 ± 1.3 | 0.13 ± 1.55 | 1 |
| Lack of skills | 1.3 ± 0.5 | 0 ± 0.63 | 2.1 ± 1.6 | -0.25 ±1.28 | 1 |
| Lack of facilities or space | 1.7 ± 1 | 0.17 ± 1.6 | 2 ± 1.6 | -0.5 ± 1.85 | 0.65 |
| Lack of knowledge on how to exercise | 1.3 ± 0.5 | 0.17 ± 0.41 | 2 ± 1.2 | -0.13 ± 0.64 | 1 |
| Lack of good health | 1.8 ± 1 | 0.17 ± 0.41 | 1.5 ± 0.8 | 0.25 ± 0.71 | 0.81 |
| Fear of injury | 1.3 ± 0.8 | -0.33 ± 0.82 | 1.5 ± 1.4 | 0.13 ± 0.35 | 0.53 |
| Participants ranked 15 items on a 5-point Likert scale from “never” to “very often” limiting physical activity. Overall barrier score and each item score were collected. Baseline and change (post-intervention) in outcome measures for control group and intervention group. Values are means ± SD. | | | | | |
